# Medical Heresies—Anesthesia in Natural Labor, Etc.

**Published:** 1849-10

**Authors:** E. B. Haskins

**Affiliations:** Clarksville, Tennessee


ART. IV.—*Medical Heresies—Anesthesia in Natural Labor, etc.* By Dr. E. B. HASKINS, of Clarksville, Tennessee.

Simultaneous with the birth and maturation of experimental philosophy, has gradually died the spirit of disputation with the cultivators of science. Each laborer in the great field of research is content to spread before the scrutinizing gaze of his fellow-laborers, the result of his own efforts, and abide, with a becoming spirit of confidence, their impartial verdict. Enlightened by the history of the past, as to the insufficiency of argument, to sustain assumed facts and false theories, and embued with a love of truth and consistency, and trusting confidently to the honesty, intelligence, and acumen of the learned, they seldom bring to bear the batteries of *logic* upon the candid and temperate criticisms of their co-laborers.—The original mind is too intent upon the discovery of new facts, and the development of new principles, to waste its time upon a single discovery or invention. Having brought it to light, matured and perfected it, and made it comprehensible to all, it is thrown upon the world to abide its fate. If it be true and valuable, it will win its way to favor; and if it be false, the sooner it is consigned to oblivion the better.

However true this may be as a general remark, we occasionally meet with exceptions. Once in a while, we witness a great mind, in every way deserving the respect and confidence of the learned, when not infatuated by the intoxicating thought of originality; yet when its dogmas are objected to, at once assumes the attitude of the combatant, and defends its favorite theory or hypothesis with the reckless anxiety of a sworn partizan—blinded by the prejudices of authorship, it brings into requisition even the weapons of false and sophistical reasoning.

An example of such a misdirection of learning and talents, may be found, in my humble judgment, in the attempt of Prof. J. Y. Simpson, of Edinburgh, to urge upon the medical profession, by the force of his genius, the adoption of anesthetic agents in *natural labor.**

In this attempt, the Edinburgh Professor has been driven, by the forcible objections of Prof. Meigs to anesthesia in *natural labor*, to the advancement of certain doctrines, that, I am persuaded, are new to the profession, and never would have been advanced but to sustain the consistency of a practice, with which the Professor has identified much of his medico-literary reputation; and it is the object of this paper briefly to inquire, *directly*, into the truth of these doctrines, and *incidentally*, into the propriety of anesthetic agents in natural labor.

For the sake of convenience, these doctrines may be stated in three propositions, in the order in which they are argued by Prof. Simpson :†

---

* This will scarcely be said to be too strong a charge, when his own countryman and eulogist holds the following language about him: "No sooner was his anesthetic system impugned, than Professor Simpson threw himself tooth and nail into the conflict; and his adversaries, after experiencing about as severe punishment as men could stand up and receive, are now beginning to understand their position."—*Eclec. Mag. of For. Lit.* from *Tait's Edinburg Magazine.*

† See Prof. Simpson's Letter to Prof. Meigs, published in the Med. Examiner for April and May, 1849.

*Prop. 1st. That pain is a non-essential element in the process of parturition.*

*Prop. 2d. That the pains of parturition are not physiological, in civilized society.*

*Prop. 3d. That physiological functions may be proper objects of therapeutic interference.*

The argument to sustain the first proposition was as follows: "It [the pain of labor] is non-essential because, first, labor, that is, the uterine contractions, are occasionally, though very rarely in the course of practice, seen to accomplish the full expulsion of the child, with little or no pain. Second, in whole tribes of the human race, as in some of the black tribes, little or no pain seems to be endured, if we may believe various authorities; and third, hundreds of women have, during the last year, been delivered, with perfect safety, but without any pain, while placed under the influence of anesthetic agents."

Here it seems that the Edinburg Professor has strangely overlooked the difference between that which is essential to the mere performance of an act, and that which is essential to its best or complete performance—between that which is not absolutely essential and that which is more than essential, or superfluous. Two eyes are not essential to the mere function of vision, yet that is no reason why one should be plucked out as *non-essential*, or superfluous. Two eyes are essential to complete or perfect vision—just as pain may be affirmed to be not essential to the mere act of parturition, yet essential to perfect parturition. Besides, it may even be true, that pain is not essential to the perfect expulsion of the child, and yet not true that it is a non-essential element in the process of parturition; for the human constitution is a system of organs, intimately connected by functional relationships, many of which, no doubt, being feebly exerted upon distant organs, are wholly unknown to us; and though the pains of labor may seem to serve no essential

2 *

end in the perfect expulsion of the child, yet have some other salutary influence not cognizant to our senses.

The laws of organic action are not sufficiently understood, and probably never will be, to justify us in the declaration of any physiological action, that it is non-essential, *simply because we may not perceive its essentiality.* Such a facile disposition of physiological problems will hardly meet with the approbation of the rigid and exact philosophers of the present day. It may not be uninteresting to show, that by the same mode of reasoning as here instituted, to prove the non-essentiality of pain in the process of parturition, the feet can be proven to be non-essential to the " process of progression,"* and therefore may be dispensed with. The argument will run thus: Feet are non-essential elements in the " process of progression, because 1st, the legs are occasionally, though very rarely, in the course of life, seen to accomplish the process of walking with little or no feet. 2d. In whole tribes of the human race, as in the Chinese Empire, *little* or no feet seem to be possessed, if we may believe various authorities; and 3d. Hundreds of persons have been seen performing the act of progression with ease, but without any feet, while placed on crutches, or upon wooden legs.

By the way, the *little* and *no*, in the Professor's argument, are ingeniously tacked together, and are, *by turns*, important and *independent* words in the argument. The " little" is important to the truth of the statement, that " in whole tribes *little* (or no) pain is endured," and the " no" becomes important and necessary to the legitimacy of the conclusion *that therefore pain is non-essential*—why? Because " (little or) *no* pain is endured."

I will here take occasion to deny such a thing as a nat-

* I acknowledge my indebtedness to Prof. Simpson for the illustration.

ural delivery without pain. I presume it would scarcely be said by any one, in common parlance, that persons enjoying health, suffered any pain in passing urine and feces —yet in the exactness of scientific language, pain accompanies every voluntary effort to expel the contents of the bladder and rectum. The pain being slight, does not render it *no pain*, nor make it *non-essential* to its office.

It may be argued in direct proof of the essentiality of pain in the process of parturition, that in the second stage of labor, the voluntary contraction of the diaphragmatic and abdominal muscles are also brought into requisition in the expulsion of the fetus; and although it may be granted that the agency of these muscles are mainly referred to the laws of reflected and consentaneous action, yet it will hardly be denied by any midwife, that the act of volition greatly controls and modifies these contractions.

If it should be objected that the pain which excites these voluntary contractions is not the pain of uterine contraction, but that arising from the action of the fetus upon the sensitive vaginal walls, it may be replied, that that does not affect the argument as applied to the doctrine of anesthetic agents, since to annul the uterine pains, will also be to annul the pains of original distension.

*Prop.* 2. *That the pains of parturition are not physiological, in civilized society.*

The argument is as follows: " Now, for the reason that I have already stated, I entirely doubt if we should look upon the severe sensations of pain endured by our patients as truly ' physiological' for, as I have just stated, they are not essential to the mechanism and completion of the process in the white races of mankind, and they are absent to a great degree in the black. The severity of them could, I think, be easily proved to be the result of civilization, and, as I believe, of that increased size of the infantile head, which results from civilization."

Having already examined the arguments upon the non-essentiality of pain in labor, it remains only to inquire into the soundness of the position that *severity* of the pain necessarily constitutes it *pathological*, instead of *physiological.*

It is somewhat surprising that Prof. Simpson should have overlooked a principle recognized by all physiologists, that the functions of the animal organism may and constantly do undergo augmentations and depressions, without passing beyond the physiological limits; thus, the range of healthy urinary secretion, is, according to M. Rayer, between twenty-one and fifty-seven ounces of urine in the twenty-four hours. Andral estimates the physiological range of fibrine of the blood to be between two and five-tenths and three and five-tenths, and that of the globules between one hundred and ten and one hundred and forty; and so with every other functional product, no doubt, in the human body.

That the pains of labor, as well as of hunger, thirst, and every other physiological sensation, may, and actual ly do have some limits, beyond which they become pathological, may be granted, and yet it does not follow that the severe pains of natural labor—pains that are severe, *ex necessitate*—are pathological. Of course, the enlightened Accoucheur is the proper judge of the pathological or physiological nature of *each particular case that is committed to his care*, as well as of the *safest and best means of relieving them*, or bringing them within the *physiological limits.*

Again, I presume it would scarcely be doubted by any one, that the various modes of artificial life, consequent upon civilizatiion do augment certain functions and depress others, and at the same time the constitution, from its wonderful power of adaptation to the various conditions and circumstances of life, preserves the healthy balance. To take for granted, as Professor Simpson seems to have done, that that augmentation or depression of physiological action which is the result of *civilization*, is necessarily pathological, would be to deny a healthy action to almost every organ in the constitution of persons of civilized life. Indeed, the Professor has been unfortunate enough to furnish, in the same sentence, the best refutation of his own position, in admitting that *the increased size of the infantile head is the result* of *civilization*, for I imagine that he will not contend that the *increased head is a diseased growth*, or the *result of a pathological action.*

But the Professor, with seeming consciousness of the difficulty of sustaining such a proposition, ingeniously waives the further discussion of it, and directs the force of his logical powers to the establishment of

*Prop.* 3. *That physiological functions may be proper objects of therapeutic interference.*

"But waiving this point, or the discussion of it, let me state that even if I allowed all the intense pain of parturition to be 'physiological pain,' I cannot conceive that to be any adequate reason for our not relieving women from the endurence of them. Because nature has fashioned any particular physiological function in any particular manner, that, I opine, is no reason why the science and art of civilized life should not, when possible, alter and amend its workings. If it were improper for us, for instance, to intermeddle with the functions of the hair of the head, or of the skin generally, then all hats and other coverings for the scalp, all clothing and coverings for the body, should be at once abandoned and unconditionally condemed. If it were improper for us to alter and amend the functions of the eye, then all optical glasses, the telescope, the microscope, &c., must be thrown aside."

To "alter and amend" a physiological function, strikes me as a singularly awkward expression for one of Prof. Simpson's learning. I understand a *physiological function*

to be *the proper or healthy action of an organ,* and to change or alter that action, would be to make it something else than the proper or healthy action—i. e., *an unhealthy, or pathological action.* In other words, *altered physiology is pathology.* An *organ*, by changing its relations and imposing upon it new duties, may be altered, as skin may be converted into mucous membrane, and *vice versa*, and the *function* alters with the *organ;* but that is not altering the *physiologicnl function* of skin, or of mucous membrane; for it is no longer *skin* when changed to *mucous membrane;* nor *mucous membrane* when changed to *skin.*

It is true, the intention of medication is to alter, directly, some physiological action, and make it, for the time, pathological, as, emetics are intended to disturb and alter the physiological action of the stomach; purgatives that of the stomach and bowels; opiates to alter the physiological action of the nervous system; and blister plasters to produce a pathological condition of the skin.

Indeed, it is one of the chief arts of medicine, to poison, or make pathological, certain organs or tissues, with controllable agents, to relieve the poisoned or pathological state of other and more vital organs. It is further true, that in, perhaps, every disease, certain organs assume, for a time, functionally, altered or pathological states, that are not, themselves, primarily affected, and therefore not proper objects of direct medication; but the object and end of which is to relieve some primarily diseased organ or tissue—thus, in certain deranged conditions of the chylopoietic organs, the kidneys will secrete many substances foreign to their healthy function, as sugar, albumen, biliphein, oxalate of lime, cistine, etc. The liver, skin, kidneys, mucous membrane, and indeed all secreting organs, at times, functionally, pass the physiological limits, for certain curative ends.

But, it must be observed, these alterations of *nature,*

and also of medicine, which are but the suggestions and aids of nature, are not made with the same view, that Professor Simpson proposes to "alter and amend" physiological "workings"—*not because nature has fashioned the particular physiological functions in this particular manner*, and that therefore they are at fault in their *physiological* relations; but because the *physiological* "workings" have been by some hurtful agent, *already* "altered," and the physiological harmony disturbed, and these temporary alterations are made to *restore* the physiological "workings" *that nature has fashioned after a particular manner.*

It may now be asked, what *physiological functions* of the hair of the head, skin, and eyes, do hats, clothing for the body, and optical instruments "alter and amend"? If to cover and protect the head and defend the eyes and face from the rays of the sun be the function of the hair here alluded to, and I can imagine no other, then hats do not *alter* that function, as the hair still covers the head in the *same manner;* nor do they *amend* the function, as it covers the head *no better*, by wearing hats; and the same is true of the other examples. The alterations and additions are not made upon the organic powers and forces; but upon things external of them. *Physiological functions* can only spring from *anatomical structures*, and *hats, clothing* and *optical instruments* being no parts of anatomical structures, cannot be said to perform parts in physiological functions—they may aid* and assist the function; but that aid and assistance forms no fraction of the *physiological action.*

But we will pursue his argument further: "You [Prof. Meigs] talk as if supposing that we should not use means to eschew the pain of parturition, because that pain is physiological. When Columbus first discovered your

* Functions are generally *enfeebled,* instead of *improved,* by aids.

mighty American continent a large portion of the inhabitants were unprovided with any kind of dress or covering. 'To most of them,' says Robertson, 'nature had not even suggested any idea of impropriety in being altogether uncovered.' And I do think that men living in such a state could, against the fashion of dressing, use with far greater propriety and consistency than you or I your own argument against anesthetics in labor." * * * * * "The truth is, all the tendencies of man in a civilized state of society are to imtermeddle with and change, and, as he conceives, improve the action of almost every function in the body. And each such improvement has, at the time of its introduction, been, like the practice of anesthesia, very duly denounced as improper, &c. I might refer to numerous such cases. Let me cite only one example. The human fingers are admirably constructed by our Creator for the function of seizing and lifting objects." * * * "In the reign of the earlier Stuarts, forks were introduced from the continent to assist our hands in the act or function of seizing and lifting the divided portions of meat, &c., that we wished to eat. But this was a very sad and uncalled for innovation upon the old and established physiological functions of the human fingers; and at the time it was as loudly opposed and descried as the modern employment of anesthetics in aiding the physiological function of human parturition." [That anesthetics *aid* the physiological function of parturition is, virtually, an assumption of the thing in dispute.] * * * * * "I repeat, all our tendencies and workings in the present state of civilization, are attempts to intermeddle with and change and improve the action of almost every function in the economy. And assuredly, if we use means in regard to the function of parturition, with a view of ameliorating the unnecessary, but, as you call them, 'absolutely indescribable' pains that attend upon it, we would be doing nothing more than what you and I and all of us are ever doing, in most of the other natural or physiological functions of our bodies. Let me illustrate this last remark by one more example, for, as I have already said, it is only in this way that we can properly judge of the soundness or unsoundness of our views of novel points in theory and practice. You are well aware that the act of parturition has often been familiarly compared, as the late Prof. Hamilton expresses it, 'to the toils of a journey, and, like it, divided into stages. 'The sufferings of the mother (says he) have been in most languages compared to those of travellers.' Now let us, for a moment, consider this natural simile between the function of parturition and the function of progression. You maintain that 'labor is the culminating point of the female somatic forces.' One of the most illustrious Presidents of your great American republic—Thomas Jefferson—makes, in his memoir, a remark of precisely the same import, regarding walking or progression. He describes the act of walking, but not exactly in the same words, as the kind of culminating point of the human somatic forces." Few or none, perhaps, will question the abstract truth of Jefferson's observations on this point. But, because walking or progression is a 'physiological' function, and the practice of it reputed salutary, would this be with you a proper and sufficient reason for never setting aside, or superseding in any way this physiological state, in the same way as you insist, on the same grounds, that the physiological pain of labor should not be set aside or superseded? Because progression is a natural condition, would this be any adequate reason for your medical advisers adopting your own arguments against anesthesia in midwifery, and insisting upon this, that the next time you traveled from your own city of Philadelphia, to the cities of Baltimore or New York, you should walk the distance on foot, instead of travelling it by railway or other conveyance?"

The learned Professor, it will be observed, has argued through these entire extracts as though it were quite a conceded point, that whatever the arts of commercial, social and fashionable life, find useful, convenient, and agreeable, for these ends, are likewise best for the ends of *hygeine* and *medicine*. That the same "intermeddling" with the functions of the constitution practiced by merchants, navigators, artists, and the fashionable, in their respective pursuits, may and ought to be practiced by the medical man in the direct discharge of a professional office. Traveling in steamboats, steamcars, and coaches, promotes the activity and perfection of commercial and social intercourse ergo, traveling in steamboats, steamcars, and coaches, promotes the best interest of the organic constitution. Thus the Professor argues. But he has prepared the reader's mind for this concession, by artfully assuming that the "intermeddling" with the functions of the body *improves* them. "The truth is, the tendencies of man in a civilized state of society are to intermeddle with, and change, and, as he conceives, improve the action of almost every function in the body."

Now we have furnished to us two examples of this *intermeddling with*, and *improvement of*, the functions of the human constitution, and it is presumed that Prof. Simpson has selected the best examples to illustrate his subject.

The examples are, 1st. The use of forks in seizing and lifting the divided portions of meat, &c. 2d. The use of carriages and other vehicles, as improvements upon the function of progression, or walking.

Allowing that seizing and lifting divided portions of meat with the fingers, and walking, be *physiological functions*, it would even then be no slight task to make it appear that these functions are *improved* by the means here specified. In what manner is the function of "seizing and lifting," by the use of forks, cultivated and improved? Are fingers more dexterous in "seizing and lifting divided portions of meat," &c., now, than before the introduction of forks? In truth, is not the function of the fingers rather superseded? And are not the same interrogations applicable respecting the function of walking? Can any one show, in what way the function of *walking* is improved by *riding* in carriages and steamcars? The *intermeddling* is not disputed; but to make out the *improvement*, we will have to assume (with Prof. Simpson?) that the parts performed by the forks, carriages, etc., constitute parts of *physiological functions*, the monstrous absurdness of which requires no further notice than has already been taken.

The Professor further argues, as though the tacit agreement of the medical profession to the various pursuits of civilized life be an implied acknowledgement of the tendency of those pursuits to the *improvement* of those *functions* of the body impressed by them. In other words, that if certain functions of the body be enfeebled by the arts of civilized life, then the medical profession should advise that they "be at once abandoned and unconditionally condemned." But this by no means follows: *Individuals*, when engaged in the various specific pursuits of life, attend especially to those objects that are to advance and perfect the ends for which they respectively labor. *Human society*, or *mankind collectively*, however, when operating in view of the interest of the whole, require each member to yield so much of the interest of his special pursuit as is necessary to the general good. Special pursuits, when exercised with a due deference to the interests and just ends of other pursuits, and consequently the interest of the whole, give aid and assistance to the progress of society; but when exercised without such a respect for the general welfare, they become retarders, instead of promoters of man's highest destiny. Therefore, the medical profession cannot be rightfully exercised in opposing the progress of commerce, agriculture, civil government, the arts, and other laudable pursuits simply because these pursuits may weaken some organs of the physical constitution, and engender disease. The medical man, in the lawful and just capacity of his profession, cannot say to society, you should not wear clothing for the body, because it enfeebles the action of the skin; and that you should not wear hats, because they usurp the function of the hair of your head;—that steamboats, steamcars, and carriages, should not be traveled in, because they indulge man in habits that enfeeble the muscular system, &c. I imagine the physician, who presumed upon this professional right, would be answered something after this manner: Sir, the human capacities, moral, mental, and social, are such as to create a great variety of wants, to answer all purposes of his being; and, therefore, he has more interests to secure than that which it is the office of your profession to subserve. To live long, with a vigorous constitution, is, certainly, of the highest importance; but when purchased at the price of every thing that ministers to his moral, mental, and social wants then it ceases to be desired by man. Your ancient and honorable profession, therefore, can serve the world better than to oppose the progress of civilization. If the various modes of artificial life, consequent upon civilization, do generate new sources of disease and death, it is expected of your profession to advance, *pari passu*, with the sister arts and sciences;—to study the etiology and nature of those diseases, that you may direct us, in our pursuits, to the best means of shunning them, and when they do arise, to apply your art to their cure. This would, I imagine, be the answer to the exercise of such an assumed professional prerogative.

In order to present more strikingly the unsoundness of the third proposition, it was my intention to discuss, at some length, the *therapeutic* and *prophylactic* relations of pain; but my space will only admit of a few general remarks upon that subject; which is, in itself, sufficiently full and interesting for a separate paper.

*Pain*, as a physical phenomenon, may very properly be considered under two heads—pathological and physiological.

*Pathological pain*, I understand to be pain that indicates a diseased or pathological state of some organ or tissue, organic or functional; and for the cure of this pathological state, and consequently the relief of the pain, there is no certain infallible remedy, that addresses itself to our instincts, or to our reason; that there is nothing in the nature of the pain, or in the disease causing it, that suggests any certain specific for its relief. In short, between the *disease* and the *cure* there is known no constant legitimate therapeutic relation. However true these remarks may be, as applied to our instinct and our reason, yet they are not strictly so, as applied to the organism; for notwithstanding our incompetency to properly discern and fully appreciate the indications of cure set up by nature, yet that such efforts are made, and often too successfully, no one denies; but the positive and relative value of these efforts are at present but little understood. Indeed, it has only been by a long course of patient observation upon disease, and the natural indications of cure, that the cultivators of medicine have discovered certain remote relations between the cure of disease and the effects of therapeutic agents. But so imperfect is our knowledge of these efforts of nature, and the power and extent of remedial agents, that it is a matter of unquestionable certainty, that the medical man may, and often does, by unwise and untimely *intermeddling* with the healthy efforts of the system, do great harm, when even in the laudable office of treating a *pathological* state.

*Physiological pain*, on the contrary, is, pain indicating certain conditions of certain organs—conditions, not diseased, but healthy, yet liable soon to become so, if the physiological demands be not promptly attended to, and the pain thereby relieved. But the remedies for this pain, unlike the remedies for the former, are *specific* in their nature, and *certain* in their effects; they require no labored research, or attentive observation for their discovery, but they are at once suggested to our instincts; they require no therapeutic skill for their application, but only the necessary conditions; and in their effects, they are not therapeutic, but prophylactic.

Hunger, thirst, the sensations that move the powers of volition in the acts of respiration, defecation, and urination, are physiological pains, and simply to mention them is to convince the reader of the truth of what is here stated. Food, water, atmospheric air, the evacuation of the rectum and bladder, are the several specific remedies for the pains here enumerated.

In like manner, the pain of natural labor being physiological, has its own specific remedy—the evacuation of the contents of the gravid uterus. All these pains, however, have their physiological limits, beyond which they become pathological, and require the aid of therapeutics: thus, hunger, and thirst may be morbid, and no degree of ingestion of food, or amount of drink, will relieve the craving appetite, or thirst; the frequent ineffectual desire to pass urine and feces, that accompanies the irritated or inflamed bladder and rectum, are further examples of pain overstepping the physiological bounds. So the pain of parturition may, also, in degree or duration, or both, become pathological, and require treatment; but as already observed, of this, the accoucheur is the only proper judge in each particular case; but in *no case*, let it be borne in mind, is *therapeutical* assistance demanded whilst the pain is simply *physiological.*

July, 1849.

